# Cloning and characterization of enoate reductase with high β-ionone to dihydro-β-ionone bioconversion productivity

**DOI:** 10.1186/s12896-018-0438-x

**Published:** 2018-05-09

**Authors:** Xuesong Zhang, Shiyong Liao, Fuliang Cao, Linguo Zhao, Jianjun Pei, Feng Tang

**Affiliations:** 1 Co-Innovation Center for Sustainable Forestry in Southern China, Nanjing Forestry University, 159 Long Pan Road, Nanjing, 210037 China; 2 College of Chemical Engineering, Nanjing Forestry University, 159 Long Pan Road, Nanjing, 210037 China; 3 College of Tea and Food Science and Technology, Jiangsu Polytechnic College of Agriculture and Forestry, Jurong, 212400 China; 4 Jiangsu Key Lab for the Chemistry & Utilization of Agricultural and Forest Biomass, 159 Long Pan Road, Nanjing, 210037 China; 5 International centre for bamboo and rattan, 8 FuTong East Street, Beijing, 100714 China

**Keywords:** β-ionone, Dihydro-β-ionone, Enoate reductases, Artemisinic aldehyde reductase

## Abstract

**Background:**

Dihydro-β-ionone is a principal aroma compound and has received considerable attention by flavor and fragrance industry. The traditional method of preparing dihydro-β-ionone has many drawbacks, which has restricted its industrial application. Therefore, it is necessary to find a biotechnological method to produce dihydro-β-ionone.

**Results:**

In this study, the enoate reductase with high conversion efficiency of β-ionone to dihydro-β-ionone, DBR1, was obtained by screening four genetically engineered bacteria. The product, dihydro-β-ionone, was analyzed by GC and GC-MS. The highest dihydro-β-ionone production with 308.3 mg/L was detected in the recombinant strain expressing DBR1 which was later on expressed and purified. Its optimal temperature and pH were 45 °C and 6.5, respectively. The greatest activity of the purified enzyme was 356.39 U/mg using β-ionone as substrate. In the enzymatic conversion system, 1 mM of β-ionone was transformed into 91.08 mg/L of dihydro-β-ionone with 93.80% of molar conversion.

**Conclusion:**

DBR1 had high selectivity to hydrogenated the 10,11-unsaturated double bond of β-ionone as well as high catalytic efficiency for the conversion of β-ionone to dihydro-β-ionone. It is the first report on the bioconversion of β-ionone to dihydro-β-ionone by using enoate reductase.

**Electronic supplementary material:**

The online version of this article (10.1186/s12896-018-0438-x) contains supplementary material, which is available to authorized users.

## Background

Ionones were widely used in daily flavor formula. Most Ionons were ionon and methyl ionone, and it cannot satisfy the market demand with weak production capacity and single variety. Dihydro-β-ionone, also called ‘sweet *osmanthus* king’, has received great attention from the flavour and fragrance industry. It is a main aroma compound with mellow, sweet, and fresh cedar scent in *Osmanthus* oil. Because of its unique scent, commercial extracts are in high demand for use in the production of expensive perfumes and cosmetics [1]. It is widely used in foodstuff and beverage industries. It is also an important intermediate compound used in the synthesis of tea screw alkanes and its analogues with great application foreground [2, 3]. Dihydro-β-ionone naturally occurs in *Osmanthus fragrans* Lour, roses, and in many flowers. For a number of ingredients, their extractions from plant or animal tissues are the best way to get the products. However, the aroma compound dihydro-β-ionone is of particular interest because it occurs mostly at low concentrations in natural tissues and extraction in most situations is not economically feasible [4]. In addition, the extraction of aroma compounds from natural source is an expensive and arduous task and strongly depends on agriculture and all the factors surrounding it [5]. The chemical production process of dihydro-β-ionone is a catalyzed hydrogenation process from β-ionone, which is performed by using asymmetric hydrogenation of costly catalysts like chiral rhodium or ruthenium phosphines [6, 7]. The limiting issues involved in the chemical production process are the disposal of complex heavy metal ligands and the requirement of high pressure [8]. Therefore, a specific branch of biotechnology has been developed in which constraints relative to the natural state are present [9].

Biotechnology represents a very attractive alternative for the sustainable production of flavors and fragrances [10]. With the increasing development of genetic engineering, it became possible to produce heterologous products in microbial cell factories that are normally found only in small amounts in nature [11, 12]. The highly demanded phenethyl alcohol, vanillin, and γ-decalactone can also be produced through biotechnology of natural products, but, for many compounds, yields are very low and the products are too expensive to satisfy demand [13–17]. In recent years, enoate reductases which are members of the ‘old yellow enzyme family’, have been attracting the interest of chemists for its high potential in asymmetric reduction of activated C=C bonds [18]. Enoate reductases have a wide variety of substrates, such as α,β-unsaturated aldehyde ketone, nitroalkenes, α,β-unsaturated nitriles, α,β-unsaturated carboxylic acids and their derivatives [19–21]. Enoate reductases have been found in plant, bacteria, fungi, and protozoa [22–25], and play critical roles not only in the biosynthesis of steroids, fatty acids, and phytohormones like jasmonic acid [26], but also in plant secondary metabolism, such as pulegone and artemisinin biosynthesis [27, 28]. In the area of enoate reductases enzymatic hydrogenation, research efforts mainly focused on whole-cell systems with wild-type microorganisms, new enoate reductases with highly catalytic activity, and broad substrate profile [29]. There exists almost no research on the conversion of natural active substance with enoate reductases, and the demand of industrial production could not be met because of the absence of high activity, stability, and tolerance to the system.

In the present study, high enoate reductases were screened and β-ionone was converted into dihydro-β-ionone by whole-cell systems. Overpression and characterization of the optimization of enoate reductases have also been reported. This enzyme has high selectivity for transforming β-ionone to dihydro-β-ionone. These extraordinary properties enable enoate reductases a good biocatalyst for producing dihydro-β-ionone in vitro.

## Methods

### Bacterial strains and plasmids

The genes encoding DBR2 (NCBI accession number BAU61367.1) from *Artemisia annua*, BacOYE1 (NCBI accession number KJ577134.1) from *Balillus* sp., DBR1 (NCBI accession number FJ750460.1) from *Artemisia annua*, and Unigene (CL2687.Contig2_ALL) from *Osmanthus fragrans* transcriptome sequencing (SRA accession number SRP057917) which shared 78% identity with the DBR2 of *Artemisia annua* (NCBI accession number BAU61367.1) were synthesized by Shanghai Generay Biotech Co. Ltd. (Shanghai, China). The synthetic genes were inserted into plasmid PGEX-4 T1 or pET-28a (Invitrogen, USA) to generate the expression vector pET-28a-BacOYE1, pET-28a-DBR1, PGEX-4 T1-DBR2, and PGEX-4 T1-2687. Then recombinant plasmids were transformed into *E. coli* BL21 (DE3). The sequence of DBR1, DBR2, BacOYE1, and CL2687.Contig2_ALL was shown in Additional file 1.

### Production of dihydro-β-ionone on whole-cell systems

One hundred microliters of an overnight culture was used to inoculate 5 ml of LB resistance medium at 37 °C to an optical density at OD600 of 0.4–2.0. The expression of the proteins was induced by adding 0–1 mM isopropyl-b-D-thiogalactoside (IPTG). Fifty to three hundred microliters β-ionone solution to a final concentration of 10 mM was added using chloroform, DMSO, methanol, ethanol and ethyl acetate as the solvents. The bacteria were further incubated at 22–42 °C for 6–27 h and gently shaken (180 rpm). One milliliter of chloroform was added to the reaction mixture as inhibitor and extractant of assay products. The reaction was measured against β-ionone control solutions under the same reaction conditions with cells carrying plasmid without insertion. Production was analyzed using GC and GC-MS.

### Expression and purification of the optimization of recombinant DBR1

For protein expression, 1 ml of an overnight culture was used to inoculate 50 ml of LB kanamycin medium at 37 °C to an optical density at OD600 of 1.8. The expression of the proteins was induced by adding isopropyl-b-D-thiogalactoside (IPTG) to a final concentration of 0.6 mM, and the bacteria were further incubated at 27 °C for 18 h and gently shaken (180 rpm). Bacteria were harvested by centrifugation at 5000×g for 20 min at 4 °C, and then resuspended in 10 ml of 1× PBS.

Cells were lysed by sonification on ice with an MS 73 sonotrode (Bandelin Electronic, Berlin, and Germany) four times for 30 s at 10% of maximal power. Cell debris was removed by centrifugation (20,000 g, 30 min, and 4 °C). The recombinant protein was purified with Ni-chelating affinity chromatography(Novagen). The recombinant protein was eluted with an elution buffer containing increasing concentrations (20, 30, 50, 75, 100, 150, 200, and 300 mM) of imidazole. The protein was examined by SDS-PAGE. The protein concentration was determined by the Bradford method using BSA as a standard. The Bradford protein Assay Kit (Sangon Biotech, Shanghai, China) was employed for the determination.

### Enzyme characterization

The enzymatic activity of the recombinant DBR1 enzyme was assayed according to the method by Muangphrom et al. [30] and modified. The activity was determined GC using β-ionone as substrate. Assays contained 0.05 M TRIS-HCl, pH 6.5, 1 mM NADPH, 2 mM dithiothreitol (DTT), 1 mM β-ionone and 300 μg of the crude recombinant protein in a total volume of 500 μl at 45 °C for 10 min. After the incubation, 1 ml of chloroform was added to the reaction mixture as enzyme inhibitor and extractant of assay products. The enzymatic reaction was measured against β-ionone control solutions under the same reaction conditions while proteins were heat deactivated (100 °C, 10 min) prior to the incubation experiments.

The optimum temperature for enzyme activity was determined by standard assays ranging from 20 to 50 °C in the 0.05 M TRIS-HCl at pH 7.5. The optimum pH for DBR1 activity was determined by incubation at 45 °C for 10 min in a 0.05 M TRIS-HCl from pH 5.0 to 9.0. The results were expressed as percentages of the activity obtained at either the optimum pH or the optimum temperature.

To determine the effect of temperature on the stability of DBR1, the enzyme was pre-incubated in the 0.05 M TRIS-HCl (pH 6.5) for various times at 40, 45 and 50 °C in the absence of the substrate and other cofactor. The activity of the enzyme without pre-incubation was defined as 100%. The pH stability of the enzyme was determined by measuring the remaining activity after incubating the enzyme at 40 °C for 1 h in the 0.05 M TRIS-HCl from pH 5.0–9.0. Then, the residual activity of the enzyme incubated at variant pH was determined, immediately.

The effects of metals and chemical agents on the pure DBR1 enzyme were determined. Fe^3+^, Ca^2+^, Na^+^, Li^+^, K^+^, Mg^2+^, Zn^2+^, Al^3+^, Fe^2+^, NH_4_^+^, Mn^2+^, Cu^2+^, Ba^2+^, Hg^2+^, Co^2+^, Sr^2+^, Fe^2+^, DTT, and NADPH were assayed at the final concentrations of 1 and 5 mM in the reaction mixture. The control reaction was measured against under the same reaction conditions while DTT was absence. The effects of organic solvents on the enzyme were determined by adding 1, 2, 3, 4, 5 and 6% organic solvents (ethanol, methanol, and DMSO) to the reaction mixture.

The kinetic constant of DBR1 was determined by measuring the initial rates at various β-ionone concentrations (0.2, 0.4, 0.6, 0.8, 1.0, 1.2, 1.4, 1.6, and 1.8 mM). The reaction conditions were activated by 15 μg purified enzyme protein under standard conditions.

### Analysis of GC and GC-MS

Dihydro-β-ionone was analyzed using GC 6890 system (Agilent, USA) and a HP5 column (30 m × 0.25 mm × 0.25 μm) with flame ionization detector. Injection and detector temperatures was 250 °C, Helium is employed as the carrier gas with the flow rate at 1 ml/min. Sample size and flow were 0.2 μl and 2 ml/min, respectively. Temperature-rising program was 50 °C for 2 min, raising to 120 °C at 10 °C/min for 2 min. Then the temperature was raised to 200 °C at 5 °C/min for 5 min. Dihydro-β-ionone was also verified using Trace ISQ-LT GC-MS (Thermo Fisher, USA) and a DB-5MS column (30 m × 0.25 mm × 0.25 μm) with flame ionization detector. Interface temperature was 250 °C, EI-MS with 70 EV ionization energy and swath range were 50–450 m/z.

## Results

### Analyzing the sequence of four cDNA clones

**Fig. 1:**
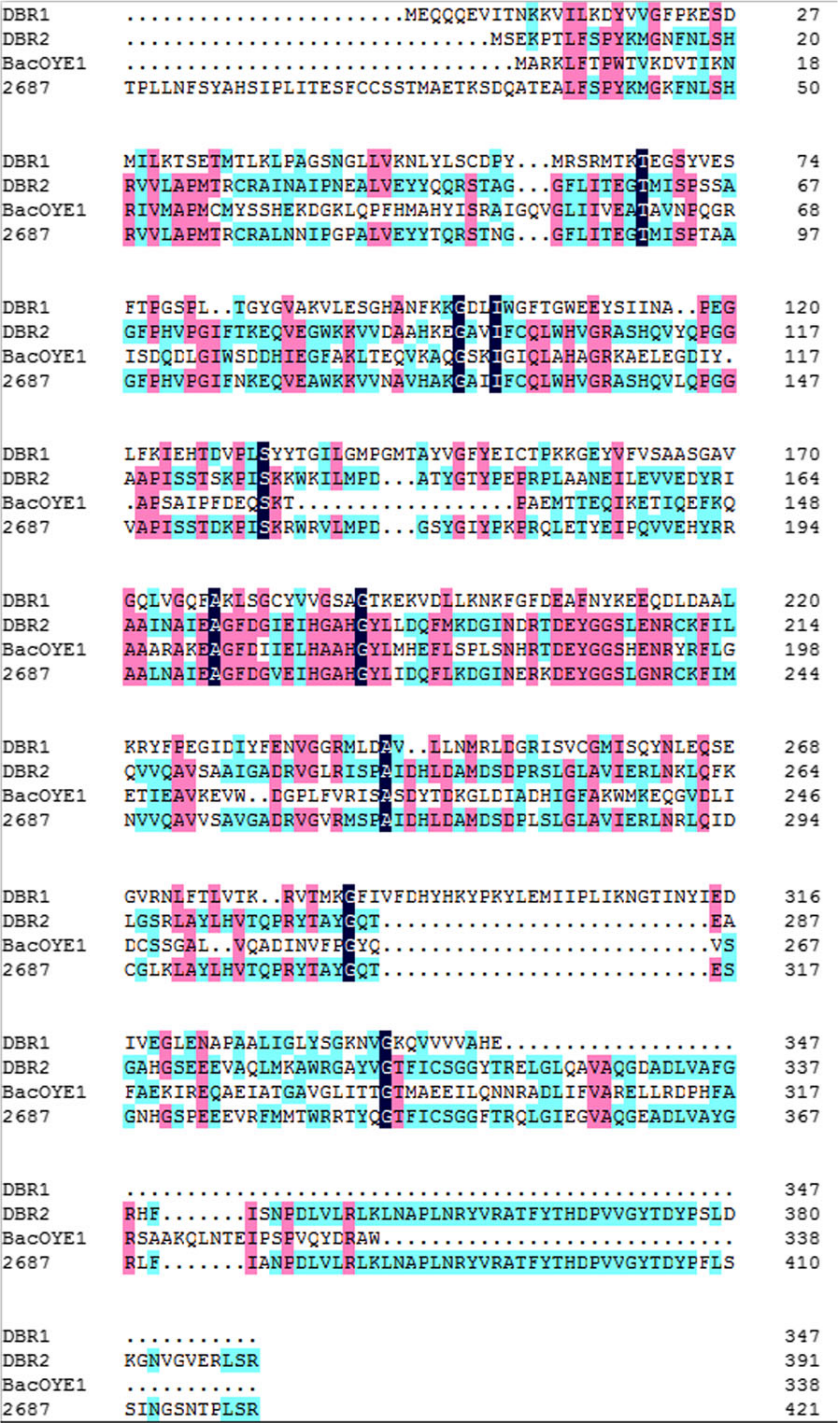
Comparison of the sequence four clones by using Multi-alignment. Multiple sequence alignment was performed by using Clustal X1.8. Full species names and Genbank IDS of four clones are as follows: DBR1, *Artemisia annua*, FJ750460.1; BacOYE1, *Balillus* sp., KJ577134.1; DBR2, *Artemisia annua*, BAU61367.1; 2687, Unigene (CL2687.Contig2_ALL) from *Osmanthus fragrans* transcriptome sequencing (SRA accession number SRP057917)

Four cDNA clones were identified as potential candidates. Based on BLASTP searches of public databases, amino acid sequence of four clones were identified with Δ11(13) reductase, 12-oxophytodienoate reductases and enoate reductases enzymes, both of which have high potential in asymmetric reduction of activated the double bonds. Sequence analysis indicated that DBR2, BacOYE1, and 2687 share some conserved motifs (Fig. 1): EAGFDG (residues 171–176), EIHGAHGYL (residues 178–186), and DEYGGSLENR (residues 200–209). Unlike other three cDNA clones, DBR1 has own characteristics: it has a glycine-rich motif, AASGAVG (residues 165–171), which is known to participate in binding of the pyrophosphate group of NAD(P)H or NAD(P)+ [31]. In addition, a Tyr in the substrate binding site of Dbr1 (Tyr59) and a putative catalytic Tyr of Dbr1 (Tyr262), in the conserved domain “SQY”, were not find in other three clones.

### Selection and production of dihydro-β-ionone on whole-cell systems

**Fig. 2:**
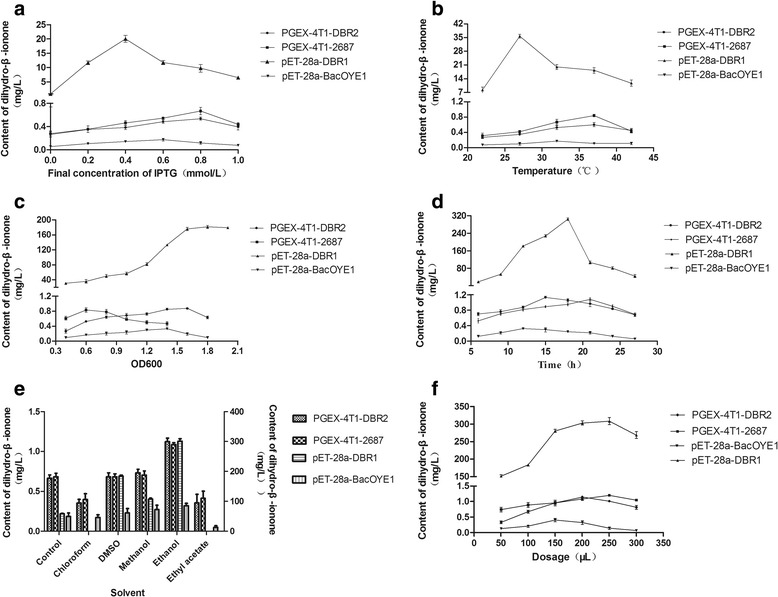
Optimization of dihydro-β-ionone preparation on the whole cell systems by four recombinant *E. coli*, namely, pET-28a-BacOYE1, pET-28a-DBR1, PGEX-4 T1-DBR2, and PGEX-4 T1-2687 against different parameters. **a** final concentration of IPTG; **b**, temperature; **c**, OD600; **d**, time; **e**, solvents and **f**, dosage (data represent the mean values of three replicates, and *error bars* represent the standard deviation)

**Fig. 3:**
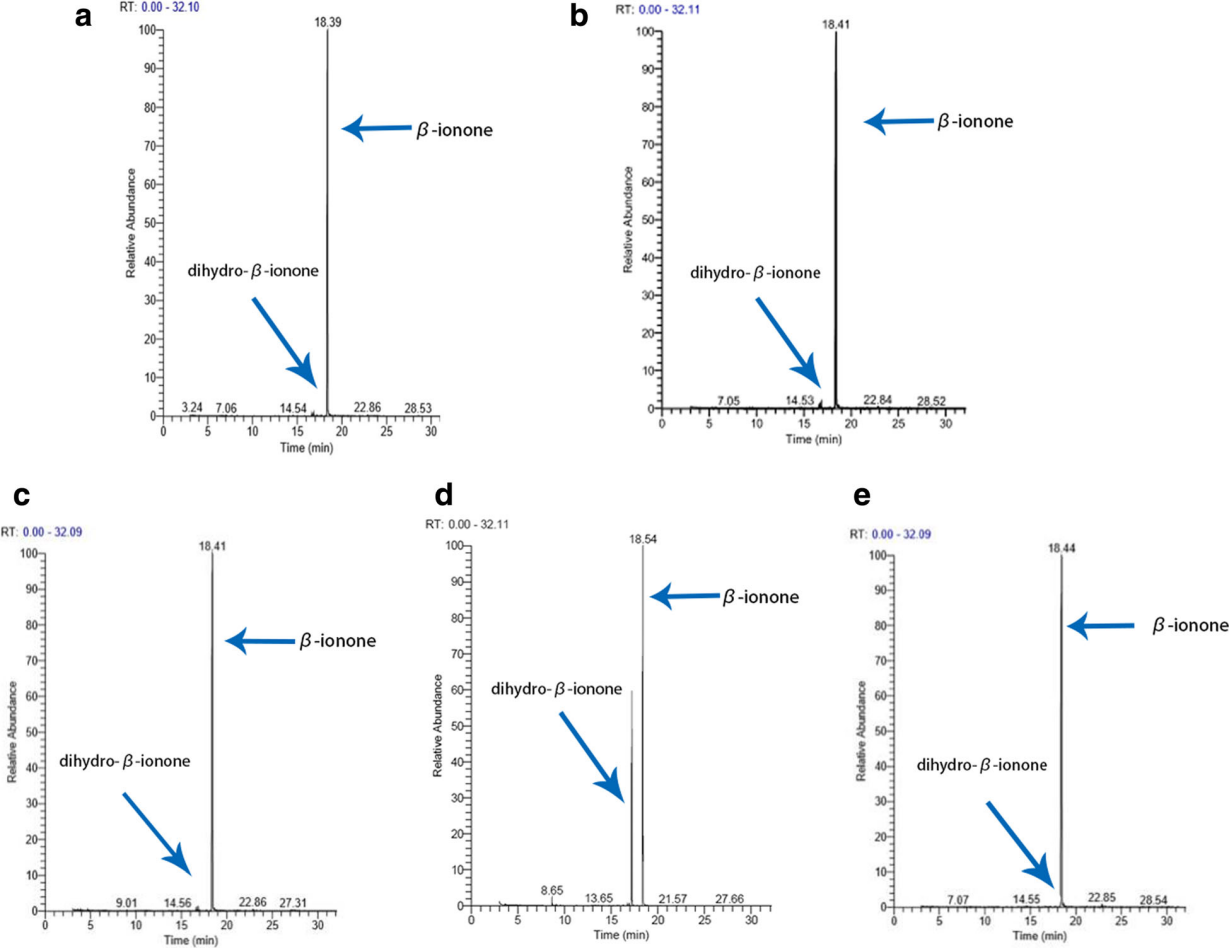
Total ion diagrams of GC-MS on the biotransformation of four recombinant *E. coli*: **a** PGEX-4 T1-DBR2, **b** pET-28a-BacOYE1, **c** PGEX-4 T1-2687, and **d** pET-28a-DBR1. **e** Total ion diagram of GC-MS on the biotransformation of cells carrying plasmid without insertion

Four genetically engineered bacteria were selected on the basis of their yield rate of dihydro-β-ionone on the whole-cell systems (Fig. 2). The recombinant strain pET-28a-DBR1 showed strong ability on transforming β-ionone to dihydro-β-ionone (Fig. 2). A total of 308.3 mg/L dihydro-β-ionone was obtained with a final concentration of IPTG 0.4 mM at OD600 1.8 and using ethanol as solvent. The addition of β-ionone in the system was 250 μl and the temperature maintained was 27 °C for 18 h. Under this condition, the yield of dihydro-β-ionone by pET-28a-DBR1 was higher by 256-fold than PGEX-4T1-2687, 270-fold than PGEX-4 T1-DBR2, and 770-fold than pET-28a-BacOYE1. Therefore, the recombinant strain pET-28a-DBR1 could be considered as potential and effective genetically engineered organism for the transformation of β-ionone. The transformation products of four genetically engineered bacteria were analyzed by GC-MS. The total ion diagram of GC-MS on the biotransformation of four recombinant *E. coli* has been presented in Fig. 3.

### Expression and purification of the optimization of recombinant DBR1

**Fig. 4:**
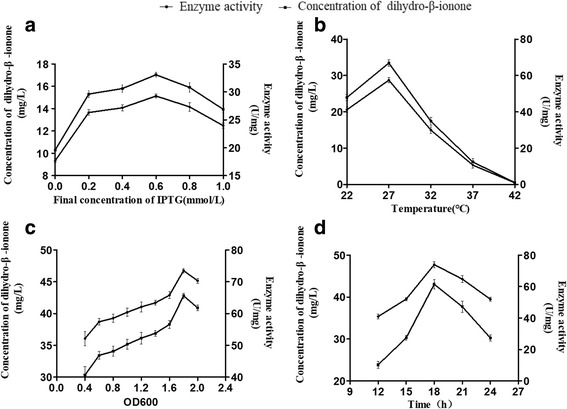
Effects of different factors on the concentration of dihydro-β-ionone. **a**, final concentration of IPTG; **b**, temperature; **c**, OD600; and **d**, time (data represent the mean values of three replicates, and *error bars* represent the standard deviation)

The recombinant pET-28a-DBR1 was transformed into *E. coli* BL21 (DE3) and was expressed for 18 h by adding 0.6 mM of IPTG and at OD600 1.8–2.0 under a temperature of 27 °C (Fig. 4). It was over expressed with an activity of approximately 73.95 U/mg whilst the dihydro-β-ionone concentration reached 43.04 mg/L. The final IPTG concentration had some effects on enzyme activity. The recombinant *E. coli* was induced at 27 °C with a final IPTG concentration ranged from 0 to 1 mM during which the activity of the enzyme activity showed a certain change. Inducing with no IPTG, 17.69 U/mg of DBR1 activity was detected in the soluble fraction, which indicated that the recombinant DBR1 could be expressed without the expressive and toxic IPTG. The unexpected expression with no IPTG may have caused due to low concentration of lactose from the LB medium [32]. DBR1 was also expressed using arabinose instead of IPTG [33]. In addition, it has also been observed that the induction temperature played an important role for the enzyme activity. DBR1 enzyme activity was found at a very low level (0.88 U/mg) if induced with a temperature greater than 37 °C. This may be caused by pellet autolysis after extensive incubation at a high temperature [34].

**Fig. 5:**
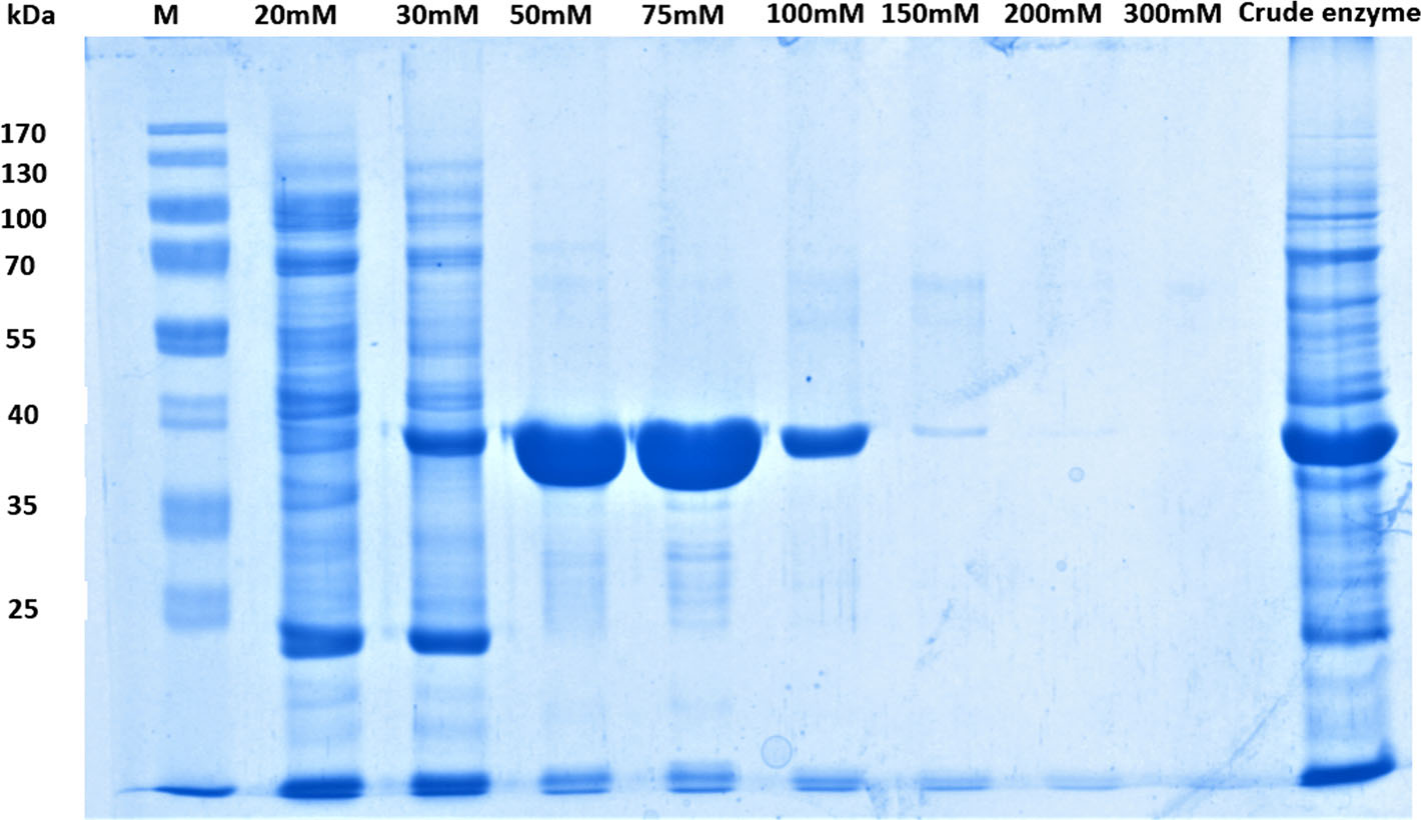
SDS-PAGE analysis of recombinant enzyme. M: marker; lane1–8: different concentrations of imidazole elution; lane 9: crude enzyme

The soluble protein and pure protein with different concentrations of imidazole elution were analyzed by SDS-PAGE (Fig. 5). The target protein was fused with His-tag, and the molecular weight of fusion protein was approximately 38.5 kDa. This finding is consistent with Zhang [33]. The majority of the reductase activity was eluted with 75 mM imidazole. The activity of purified DBR1 was 3.2-fold higher than that of the crude soluble fraction (Table 1).

**Table 1:** Purification scheme for the recombinant protein

Purification step	Total protein (mg)	Enzyme activity (U/mg)	Purification (fold)
Crude extract	22.94	112.01	1.0
Ni affinity chromatography	5.02	356.39	3.2

### Characterization of recombinant DBR1

**Fig. 6:**
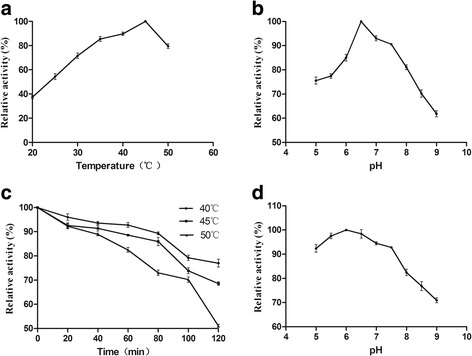
Effects of pH and temperature on the activity and stability of the recombinant DBR1. **a**, temperature; **b**, pH; **c**, thermo stability of the enzyme DBR1; **d** pH stability of the enzyme DBR1 (the residual activity was monitored, while the enzyme was incubated at 40, 45, and 50 °C; the initial activity was defined as 100%; all these activities were expressed as relative values; data represent the mean values of three replicates, and *error bars* represent the standard deviation)

The enzyme properties of recombinant DBR1 were characterized by using the crude enzyme. The optimal temperature was 45 °C, and the activity was nearly 80% of the maximum activity within a temperature range of 35–50 °C (Fig. 6a). The optimal pH determined was 6.5 (Fig. 6b). Thermostability assays of the recombinant DBR1 showed that its residual activity was almost 80% after incubating at 40 °C for 2 h, which was more than 90% for an incubation period of 1 h (Fig. 6c). The enzyme retained over 70% of its optimum at pH activity between 7.0 and 8.0. The residual activity was more than 90% of the original enzyme activity after an incubation period of 1 h at a temperature 40 °C having pH range 5.0–7.5 and in the absence of the substrate (Fig. 6d). The recombinant protein was more stable at an acidic pH and has a little different value to 2-nonenal for the substrate of DBR1 [33].

Effects of various metal cations on the activities of DBR1 were also investigated with final concentrations of 1 and 5 mM (Table 2). The activity of DBR1 was inhibited completely by Cu^2+^ and Hg^2+^, and it was also strongly inhibited by a high concentration of Fe^3+^, Zn^2+^, and Fe^2+^. On the contrary, the activity of DBR1 was increased by Al^3+^, Ca^2+^, Ba^2+^, Mg^2+^, Sr^2+^, Ni^2+^, Na^+^, K^+^, NH_4_^+^, and DTT. The catalytic efficiency of the reaction system increased significantly with Al^3+^, Ca^2+^, Ba^2+^, Mg^2+^, Sr^2+^, Na^+^, K^+^, and NH_4_^+^ added at 5 mM final concentration. DTT and Ni^2+^ showed no difference both in high and low concentrations. The activity also increased by adding 5 mM Co^2+^, Mn^2+^, and Li^+^ but inhibited at low concentration. In addition, recombinant DBR1 showed no ability on transforming β-ionone to dihydro-β-ionone when NADPH was absent. Zhang [33] compared the sequence of DBR1 with *Arabidopsis* DBR1 [35], and found a glycine-rich motif in the DBR1 sequence. This glycine-rich motif has been confirmed to participate in binding of the NADPH [31]. In the present study, monovalent cations such as Na^+^, K^+^, and NH_4_^+^, showed higher activation ability compared to divalent cations. Improved stability of NADPH might be responsible for it [36].

**Table 2:** Effects of metal cations and reagents on the recombinant enzyme activity

Cation of reagent	Relative activity		Cation of reagent	Relative activity	
	1 mM	5 mM		1 mM	5 mM
Control	100.0	100.0	Mn^2+^	78.3	114.5
Al^3+^	117.0	125.9	Hg^2+^	2.1	2.0
Fe^3+^	101.7	0.7	Ni^2+^	112.3	111.5
Ba^2+^	103.2	126.5	Na^+^	122.51	130.7
Ca^2+^	113.4	123.4	K^+^	111.7	123.1
Co^2+^	85.8	117.9	Li^+^	89.9	118.3
Cu^2+^	17.0	2.8	NH_4_^+^	110.5	129.2
Mg^2+^	108.6	123.0	DTT	126.6	125.3
Zn^2+^	106.5	37.1	NADPH	100.0	136.6
Sr^2+^	115.2	121.8	No NADPH	0.0	0.0
Fe^2+^	118.0	78.6			

Values shown are the mean of duplicate experiments, and the variation about the mean was below 3%

The recombinant enzyme activity can be improved by using organic solvent. Methanol, ethanol, and DMSO at suitable doses can improve the recombinant enzyme activity (Table 3). Compared with directly added β-ionone, by adding 5% ethanol, the recombinant enzyme activity can be increased to 151%.

**Table 3:** Effects of organic solvent on the recombinant enzyme activity

Organic solvent (%)	Relative activity		
	Methanol	Ethanol	DMSO
0	100.0	100.0	100.0
1	110.7	112.1	104.6
2	123.8	113.9	111.6
3	118.4	116.9	113.4
4	111.8	136.5	116.2
5	100.9	151.0	94.1
6	95.7	118.0	84.2

Values shown are the mean of duplicate experiments, and the variation about the mean was below 5%

The kinetic parameters of the enzyme were analyzed using β-ionone as a substrate, the *K*_*M*_ and *Vmax* values were 0.55 mM and 14.77 U/mg, respectively. DBR1 catalyzed the reduction of 2,3-unsaturated aldehydes, such as 2E-hexenal, 2E-nonenal, and artemisinic aldehyde. In the present investigation, although DBR1 showed a 10-fold higher specificity for 2E-nonenal relative to artemisinic aldehyde [33], specificity of similar strength was also shown for β-ionone relative to 2E-nonenal.

### Analysis of dihydro-β-ionone via biological catalytic hydrogenation of β-ionone

**Fig. 7:**
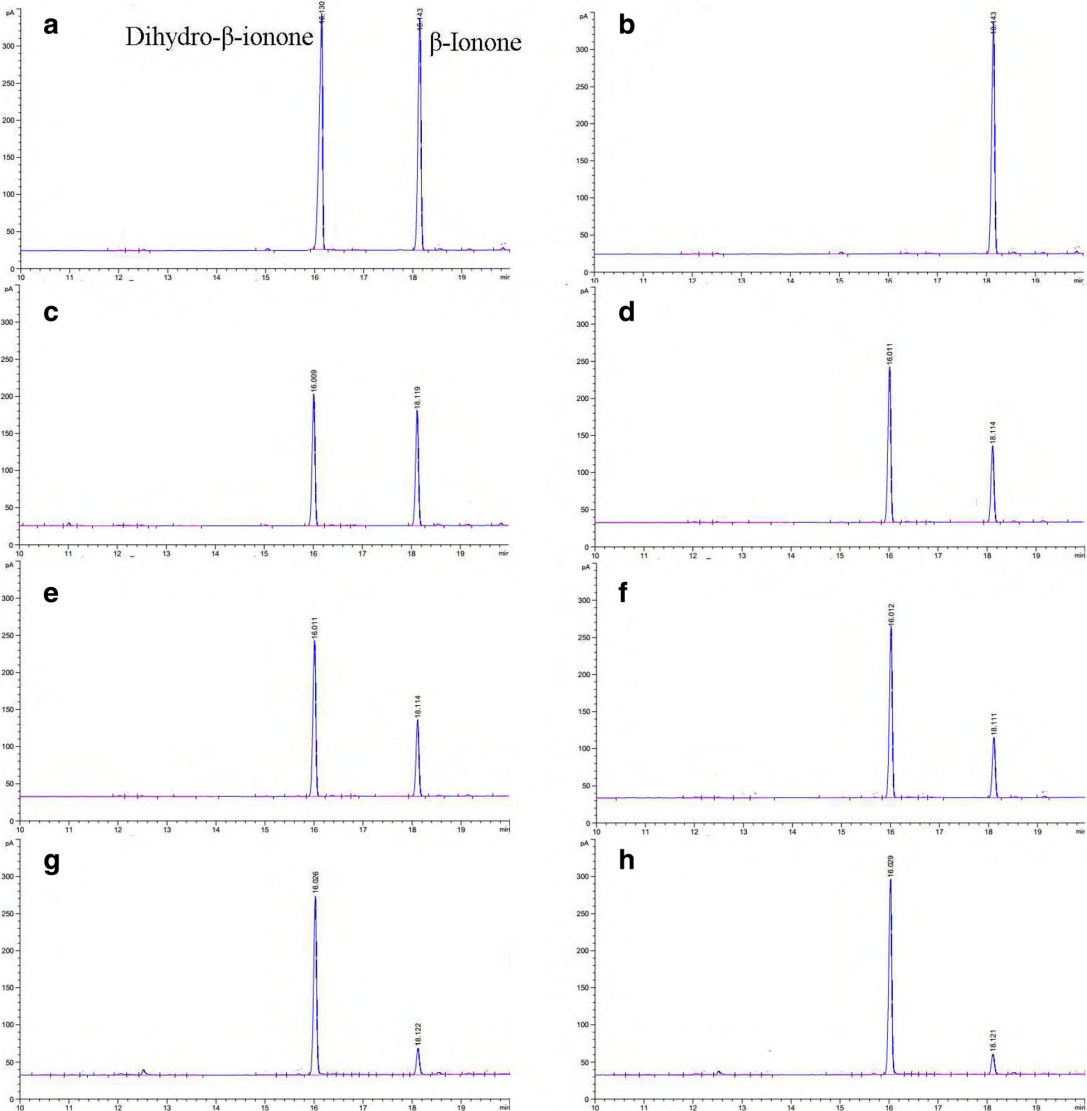
GC analysis of β-ionone hydrogenation by recombinant DBR1. **b**, **c**, **d**, **e**, and **f** β-ionone incubated for 0, 10, 20, 30, and 60 min, respectively. **g** and **h** The supplementary addition of 50 U recombinant DBR1 or 1 mmol/L NADPH after 30 min, total reaction time was 60 min. **a** Dihydro-β-ionone and β-ionone reference standard

The products of the biological catalytic hydrogenation of β-ionone were verified by GC, and a time-course experiment was performed. The reaction mixture, in a total volume of 500 μl, containing 0.05 M of TRIS-HCl (pH 6.5), 1 mM of β-ionone, 1 mM NADPH, 2 mM DTT, and 100 U/ml of the pure recombinant protein was incubated for 60 min at 45 °C. The data have been presented in Fig. 7. The maximum β-ionone concentration was found to be 76.49 mg/L (Fig. 7f), and the effective β-ionone conversion rate reached 78.77% (the biological catalytic hydrogenation of β-ionone by DBR1 enzymes led to one moles of dihydro-β-ionone per mole of β-ionone). A supplementary addition of 50 U recombinant DBR1 or 1 mM NADPH was performed after 30 min and 30 min later period of the former, so that the yield of dihydro-β-ionone can be increased to 83.12 and 91.08 mg/L. Under this condition the effective β-ionone conversion rate reached 85.60 and 93.80%, respectively.

## Discussion

In the modern synthetic biological study, the utilization of Gordian technique to invert β-ionone to dihydro-β-ionone needs the presence of an effective invertase. This task has rarely been reported. However, few publications are available regarding the conversion of unsaturated aldehydes and ketones as well as their genes. A clear distinction of substrate structure, could strongly affect enzymatic property and conversion efficiency. The enzyme employed in synthetic biology or biological catalytic conversion processes could break the boundaries of species. It could origin from the same or different plants or even may be rooted in microbes.

The artemisinin biosynthetic pathway has been studied for many years, and most of the enzymes have already been cloned [37–39]. In the artemisinin biosynthesis, artemisinic aldehyde Δ11(13) reductase (DBR) can convert artemisinic aldehyde into dihydroartemisinic aldehyde [30]. DBR1 has been cloned by Zhang [33], which can perform catalytic hydrogenation of 2,3 unsaturated aldehyde. *Artemisia annua* aldehyde and hexene aldehyde can be catalyzed by DBR1. DBR2 as cloned by Zhang [28] which has high catalytic activity to cyclohexenone and carvone. OYE homologous genes i.e., Bac-OYE1 was cloned from *Bacillus* also exhibited activities toward α,β-unsaturated aldehydes, ketones, and other α,β-unsaturated compounds [40]. In the present research, those four genetically engineered bacteria were screened to biotransform β-ionone to dihydro-β-ionone for the first time.

Dihydro-β-ionone is the characteristic aroma compounds of *O. fragrans*. There exists an enzyme in plants belonging to the genus *Osmanthus* Lour. (Fam.: Oleaceae) which could convert β-ionone into dihydro-β-ionone. Enzyme of plants might not be much effective, but it could be increasingly improved through repeated screening and research. Ro [41] published a design to assemble some genes from different organisms in order to establish non-natural metabolic pathways on yeast to synthesize largely precursor of artemisinin-artemisinic acid. This experiment simulated the natural plant’s metabolic process, to invert β-ionone to dihydro-β-ionone in vitro. Meanwhile, the present research team firstly discovered a kind of double bond reductase gene from non-*O. fragrans*, which has a strong transfer ability. Divalent cations were not necessary than monovalent cations for enzymatic activity and that DBR1 is not a metalloprotein. The DBR1 activity was not affected by DTT, which is a well-known thiol group inhibitor, suggesting that sulfhydryl groups may not be involved in the catalytic center of the protein structure. In addition, DBR1 was more stable at an acidic pH or a temperature range of 35–50 °C withβ-ionone, the cloning and characterization of Dbr1 present some biotechnological possibilities.

Based on this, in the present investigation, a recombinant strain pET-28a-DBR1 with steady catalytic performance through gene biotechnology has been achieved. This strain and its enzyme has commendable catalytic hydrogenation capacity to β-ionone’s exocyclic double bond.

## Conclusion

Of the known plants, 2-alkenal reductases substrate specificity is somewhat broad, but without a complete substrate overlap among various enzymes. In the present study, four genetically engineered bacteria were screened to biotransform β-ionone to dihydro-β-ionone and only the DBR1 from *A. annua* showed high conversion efficiency. DBR1 was also expressed, purified, and biochemically characterized. It has a high catalytic efficiency for biotransforming β-ionone to dihydro-β-ionone, and thus becomes economically more feasible. This study, therefore, demonstrates that recombinant DBR1 has great potential for industrial applications, including bioconversion for producing natural compounds.

## Additional file

Additional file 1: Sequence of DBR1, DBR2, BacOYE1, and CL2687.Contig2_ALL. (DOCX 24 kb)
