# Supplementary material for: Cloning and characterization of enoate reductase with high β-ionone to dihydro-β-ionone bioconversion productivity
Source: BMC Biotechnol. 2018 May 9;18:26. doi: 10.1186/s12896-018-0438-x (PMC5944158; doi:10.1186/s12896-018-0438-x)
Supplement: Supplementary file 1 — Sequence of DBR1, DBR2, BacOYE1, and CL2687.Contig2_ALL. (DOCX 24 kb) [file 12896_2018_438_MOESM1_ESM.docx]

**Additional file**

**Cloninging and characterization of enoate reductase with high β-ionone to dihydro-β-ionone bioconversion productivity**

Xuesong Zhang^1, 3+^, Shiyong Liao ^2+^, Fuliang Cao^1^, Linguo Zhao^1,2*^, Jianjun Pei^2,4^, Feng Tang^5^

1Co-Innovation Center for Sustainable Forestry in Southern China, Nanjing Forestry University，

159 Long Pan Road, Nanjing 210037, China

2 College of Chemical Engineering, Nanjing Forestry University, 159 Long Pan Road, Nanjing

210037, China

3 College of Tea and Food Science and Technology, Jiangsu Polytechnic College of Agriculture

and Forestry, Jurong 212400, China

4 Jiangsu Key Lab for the Chemistry & Utilization of Agricultural and Forest Biomass，159 Long

Pan Road, Nanjing 210037, China

5 International centre for bamboo and rattan, 8 FuTong East Street, Beijing 100714, China

+These authors equally contributed to this work

*Corresponding authors for Linguo zhao at College of Chemical Engineering, Nanjing Forestry

University, Nanjing, 210037, China. Phone: +86-025-85427962.

E-mail:lg.zhao@163.com.

Additional file 1: Sequence of DBR1, DBR2, BacOYE1, and CL2687.Contig2_ALL

Sequence 1：pET-28a-BacOYE1

1 CTCGAGCCAG GCACGATCAT ACTGAACCGG AGACGGAATT TCGGTATTCA GCTGTTTGGC

61 GGCACTACGG GCAAAATGCG GATCACGCAG CAGTTCGCGT GCAACAAAAA TCAGATCGGC

121 GCGATTGTTC TGTAAAATTT CTTCGGCCAT AGTACCGGTG GTAATTAAAC CAACTGCGCC

181 GGTTGCAATT TCGGCCTGTT CGCGAATTTT TTCTGCAAAG CTCACCTGAT AGCCCGGAAA

241 CACATTAATA TCTGCCTGAA CTAAGGCACC AGAGCTACAA TCAATCAGAT CCACGCCCTG

301 TTCTTTCATC CACTTGGCAA AACCAATATG ATCTGCAATA TCTAAGCCTT TATCGGTATA

361 ATCTGAGGCG CTAATACGAA CAAACAGCGG ACCATCCCAC ACTTCTTTCA CTGCTTCAAT

421 GGTTTCGCCC AGAAAGCGAT AGCGATTTTC ATGTGAGCCG CCATATTCAT CGGTACGATG

481 ATTAGACAGC GGACTCAGAA ATTCATGCAT CAGATAGCCA TGTGCGGCAT GTAATTCAAT

541 AATATCAAAG CCGGCTTCTT TGGCGCGTGC TGCTGCCTGT TTAAACTCTT GAATGGTTTC

601 TTTAATCTGT TCGGTGGTCA TTTCTGCCGG GGTTTTAGAC TGTTCATCAA ACGGAATTGC

661 GCTCGGGGCG TATATGTCGC CTTCCAGTTC TGCTTTACGA CCGGCATGGG CTAACTGAAT

721 GCCAATTTTG CTACCCTGGG CTTTAACCTG TTCGGTCAGT TTGGCAAAGC CTTCAATATG

781 ATCATCGCTC CAAATACCCA GATCCTGATC GCTAATGCGA CCCTGCGGAT TAACGGCGGT

841 GGCTTCCACA ATAATCAGGC CCACCTGACC AATTGCACGA GAAATATAAT GTGCCATGTG

901 AAACGGCTGG AGTTTACCAT CTTTTTCATG ACTACTATAC ATACACATCG GGGCCATAAC

961 AATGCGATTT TTAATGGTCA CATCTTTAAC GGTCCACGGG GTAAACAGTT TCCGAGCCAT

1021 GG

Sequence 2：pET-28a-DBR1

1 CTCGAGTTCA TGTGCAACCA CCACCACCTG TTTGCCAACA TTTTTGCCGC TATACAGGCC

61 AATTAAGGCG GCGGGGGCAT TTTCTAAGCC TTCAACAATA TCTTCAATAT AATTAATGGT

121 GCCATTTTTA ATTAACGGAA TAATCATTTC CAGATATTTC GGATATTTAT GATAATGATC

181 AAACACAATA AAACCTTTCA TGGTCACTCT CTTGGTCACT AAGGTAAACA GATTACGAAC

241 GCCTTCGCTC TGTTCCAAGT TATACTGAGA AATCATGCCA CACACGCTAA TGCGACCATC

301 TAAGCGCATA TTCAGCAGCA CGGCATCCAG CATTCTACCG CCCACATTTT CAAAGTATAT

361 ATCAATGCCT TCCGGAAAAT AGCGTTTCAG GGCTGCATCC AGATCCTGTT CTTCCTTGTA

421 ATTAAAGGCT TCATCAAAGC CAAATTTGTT TTTCAGTAGG TCCACTTTTT CTTTCGTACC

481 TGCACTACCC ACCACATAAC AACCGCTCAG TTTGGCAAAC TGACCCACTA ACTGGCCAAC

541 TGCACCGCTT GCGGCTGACA CAAACACATA TTCGCCTTTT TTCGGGGTAC AAATTTCATA

601 AAAGCCCACA TAGGCGGTCA TGCCCGGCAT ACCCAGAATA CCGGTATAAT AGCTCAGCGG

661 AACATCGGTA TGTTCAATTT TAAACAGGCC TTCCGGTGCA TTAATAATGC TATATTCTTC

721 CCAACCGGTA AAGCCCCAAA TCAGATCGCC TTTTTTAAAA TTGGCATGAC CTGATTCCAG

781 CACTTTGGCA ACGCCATAGC CGGTTAACGG ACTACCTGGA GTAAAAGATT CCACATAGCT

841 ACCTTCGGTT TTGGTCATGC GAGAGCGCAT ATACGGATCA CAAGACAGAT ACAGATTTTT

901 AACCAGCAGA CCATTAGAGC CTGCCGGCAG TTTCAGGGTC ATGGTTTCTG AGGTTTTCAG

961 AATCATATCT GATTCTTTCG GAAAGCCAAC AACATAATCT TTCAGAATCA CTTTCTTGTT

1021 GGTAATCACT TCCTGCTGCT GTTCCATGG

Sequence 3：PGEX-4T1-DBR2

1 GGATCCATGT CAGAAAAACC GACCCTGTTT TCTCCGTATA AGATGGGTAA TTTTAATCTG

61 AGCCATCGCG TTGTGCTGGC CCCGATGACA CGCTGTCGCG CCATCAATGC AATCCCTAAT

121 GAGGCGTTAG TGGAATATTA TCAGCAGCGT AGTACAGCGG GCGGTTTTCT GATTACCGAA

181 GGTACGATGA TTAGTCCTAG TAGCGCCGGC TTTCCACATG TTCCGGGTAT CTTTACCAAA

241 GAACAGGTTG AAGGCTGGAA AAAAGTGGTT GATGCCGCAC ATAAAGAAGG CGCCGTGATC

301 TTTTGCCAGC TGTGGCATGT TGGTCGCGCG AGTCATCAGG TGTATCAGCC GGGCGGTGCG

361 GCACCTATTA GCTCTACGAG CAAACCAATT TCTAAAAAGT GGAAAATTCT GATGCCAGAT

421 GCTACTTACG GCACTTATCC GGAACCACGT CCACTGGCAG CTAACGAAAT CTTAGAAGTT

481 GTGGAAGATT ATCGTATTGC CGCAATCAAT GCCATCGAAG CCGGCTTTGA TGGCATCGAA

541 ATTCATGGTG CACACGGCTA CCTTCTGGAT CAGTTTATGA AAGATGGCAT CAATGATCGC

601 ACCGATGAAT ATGGCGGTTC ACTGGAAAAT CGCTGCAAGT TTATCTTACA GGTTGTTCAG

661 GCCGTGAGTG CGGCCATTGG TGCAGATCGT GTGGGTCTGC GCATCTCTCC TGCAATTGAT

721 CATCTGGATG CTATGGATAG CGATCCTCGC TCATTAGGCT TAGCAGTGAT CGAACGTCTG

781 AACAAGCTAC AGTTTAAACT GGGTTCACGC TTAGCATATC TGCATGTGAC ACAGCCTCGT

841 TATACCGCGT ACGGTCAGAC GGAAGCCGGC GCTCACGGTA GCGAAGAAGA AGTTGCTCAG

901 TTGATGAAAG CGTGGCGCGG TGCGTATGTG GGCACCTTTA TTTGCTCAGG CGGCTATACC

961 CGCGAACTGG GCTTACAGGC CGTTGCCCAG GGCGATGCAG ACTTAGTGGC CTTTGGTCGT

1021 CATTTTATCT CTAATCCGGA CTTAGTGTTA CGCTTAAAAC TGAATGCTCC TCTGAATCGT

1081 TATGTTCGTG CGACCTTTTA TACACATGAT CCAGTTGTGG GCTATACGGA TTATCCAAGT

1141 CTGGATAAAG GTAATGTGGG TGTTGAACGT CTGTCACGCC TGTAAGCGGC CGC

Sequence4: PGEX-4T1-2687

1 GGATCCACCC CACTGCTGAA TTTTTCATAC GCACATAGCA TTCCTCTGAT TACCGAATCA

61 TTTTGCTGCT CTAGTACGAT GGCCGAAACC AAAAGTGATC AGGCTACGGA AGCACTGTTT

121 TCTCCATACA AGATGGGCAA GTTCAATCTG TCTCATCGCG TTGTGTTAGC CCCTATGACA

181 CGCTGTCGTG CCTTAAACAA TATTCCGGGT CCAGCCCTGG TGGAATATTA TACACAGCGC

241 TCTACCAATG GCGGCTTTCT GATTACCGAA GGTACGATGA TTAGTCCGAC CGCCGCCGGC

301 TTTCCTCATG TTCCGGGCAT CTTTAACAAA GAACAGGTGG AGGCGTGGAA AAAAGTGGTT

361 AATGCAGTTC ATGCCAAAGG CGCCATTATC TTTTGTCAGC TGTGGCATGT GGGTCGCGCG

421 AGCCATCAGG TTTTGCAACC GGGCGGCGTT GCTCCTATCT CAAGTACGGA TAAACCAATC

481 TCTAAACGTT GGCGCGTGTT AATGCCGGAT GGTAGCTATG GCATCTATCC TAAACCTCGC

541 CAGTTAGAAA CGTATGAAAT CCCGCAGGTT GTGGAACATT ATCGTCGTGC CGCCTTAAAT

601 GCCATTGAAG CGGGCTTTGA TGGTGTGGAA ATTCATGGTG CTCACGGCTA CTTAATTGAT

661 CAGTTTCTGA AAGATGGTAT CAACGAACGC AAAGATGAAT ATGGCGGTAG CTTAGGCAAT

721 CGCTGCAAGT TTATCATGAA TGTTGTGCAG GCCGTTGTGA GCGCAGTGGG TGCAGATCGT

781 GTTGGTGTTC GCATGTCACC AGCAATTGAT CATCTGGATG CGATGGATAG CGATCCACTG

841 AGTCTGGGTC TGGCCGTGAT CGAACGCCTG AATCGCTTAC AGATCGATTG CGGCCTGAAA

901 TTAGCATATT TGCATGTGAC CCAGCCACGC TATACCGCGT ATGGTCAGAC GGAAAGCGGT

961 AATCATGGTA GTCCAGAAGA AGAAGTTCGT TTTATGATGA CCTGGCGTCG CACTTATCAG

1021 GGCACCTTTA TTTGCTCAGG CGGTTTTACA CGTCAGTTAG GCATCGAAGG TGTTGCACAG

1081 GGCGAAGCAG ACCTAGTTGC GTATGGTCGC CTGTTTATTG CGAATCCAGA CTTAGTGCTG

1141 CGCTTAAAAC TGAATGCACC ACTGAATCGC TATGTTCGTG CGACCTTTTA TACACATGAT

1201 CCGGTTGTGG GTTATACGGA TTATCCGTTT CTGAGTAGTA TCAATGGCTC TAATACCCCA

1261 CTGTCTCGCA TGTAAGCGGC CGC
